# Characterization of *mleR*, a positive regulator of malolactic fermentation and part of the acid tolerance response in *Streptococcus mutans*

**DOI:** 10.1186/1471-2180-10-58

**Published:** 2010-02-23

**Authors:** André Lemme, Helena Sztajer, Irene Wagner-Döbler

**Affiliations:** 1 Helmholtz-Centre for Infection Research, Division of Cell Biology, Braunschweig, Germany

## Abstract

**Background:**

One of the key virulence determinants of *Streptococcus mutans*, the primary etiological agent of human dental caries, is its strong acid tolerance. The acid tolerance response (ATR) of *S. mutans *comprises several mechanisms that are induced at low pH and allow the cells to quickly adapt to a lethal pH environment. Malolactic fermentation (MLF) converts L-malate to L-lactate and carbon dioxide and furthermore regenerates ATP, which is used to translocate protons across the membrane. Thus, MLF may contribute to the aciduricity of *S. mutans *but has not been associated with the ATR so far.

**Results:**

Here we show that the malolactic fermentation (*mle*) genes are under the control of acid inducible promoters which are induced within the first 30 minutes upon acid shock in the absence of malate. Thus, MLF is part of the early acid tolerance response of *S. mutans*. However, acidic conditions, the presence of the regulator MleR and L-malate were required to achieve maximal expression of all genes, including *mleR *itself. Deletion of *mleR *resulted in a decreased capacity to carry out MLF and impaired survival at lethal pH in the presence of L-malate. Gel retardation assays indicated the presence of multiple binding sites for MleR. Differences in the retardation patterns occurred in the presence of L-malate, thus demonstrating its role as co-inducer for transcriptional regulation.

**Conclusion:**

This study shows that the MLF gene cluster is part of the early acid tolerance response in *S. mutans *and is induced by both low pH and L-malate.

## Background

*S. mutans *is considered the major etiological agent of dental caries due to its strong aciduric and acidogenic capacities. During the metabolism of dietary carbohydrates and subsequent formation of acid end-products, acidogenic bacteria can shift the plaque pH to 4 or lower within minutes and can retain it at this value for up to one hour, depending on the age of the plaque biofilm [1-4]. Demineralisation of the tooth enamel caused by low pH is the beginning of caries development. To withstand these pH fluctuations and to compete with other oral bacteria *S. mutans *has evolved an effective acid tolerance response (ATR). The ATR is induced under acidic conditions and has an optimal pH between 5.5-5. Several proteomic studies showed that more than sixty proteins were involved in this response and that many of them appeared within the first 30 minutes after acid shock, whereas full induction occurred after 90-120 minutes [5-8].

General determinants are the induction of general stress proteins, the reduction of membrane proton permeability, increased glycolytic activity and a shift to homo-fermentative metabolism, resulting in elevated lactate production. Anabolic reactions are in return down-regulated, which results in slower growth and lower cell yield [6,8-10]. The concomitant surplus of ATP is used to drive the H^+^/ATPase, which leads to an increased translocation of protons across the membrane. More specific reactions that contribute to the aciduricity are e.g. the agmatine deiminase system (AgDS). Agmatine is secreted by other bacteria in response to low pH but is internalised and deaminated by *S. mutans *to ammonia and carbamoylputrescine. The latter is further decarboxylated to putrescine, yielding carbon dioxide and ATP, which again can be used for proton extrusion [11].

Another mechanism for gaining ATP is malolactic fermentation (MLF), which is a secondary fermentation that lactic acid bacteria can carry out when L-malate is present in the medium. Its biochemical properties have been studied in detail because of the considerable biotechnological interest, since it occurs after the alcohol fermentation during wine making affecting the flavour of the wine. In MLF the dicarboxylic acid L-malate is converted to L-lactate and carbon dioxide by the malolactic enzyme (MLE) in a two step reaction without releasing intermediates. Since malic acid (pKa = 3.4, 5.13) is a stronger acid than lactic acid (pKa = 3.85) decarboxylation of L-malate leads to an alkalinization of the cytoplasm. This effect is further enlarged by diffusion of H_2_CO_2_/CO_2 _out of the cell into the gas phase. The concomitant pH gradient drives the electrogenic malate/lactate antiporter and is coupled to ATP synthesis, which is used to maintain the intracellular pH more alkaline than the environment by extrusion of protons [12,13]. *S. mutans *UA159 possesses a malolactic fermentation gene cluster, that is oriented in opposite direction to the putative regulator *mleR *[14]. A homologue of this regulator was the first lysR-type transcriptional regulator (LTTR) described in Gram positive bacteria and was shown to positively regulate MLF in *Lactococcus lactis*. A seven-fold induction of L-malate decarboxylation activity and a three-fold increase of gene expression determined by a *mleR-lacZ *fusion was observed in the presence of L-malate [15]. However, in *Oenococcus oeni *malolactic fermentation activity was not enhanced by the presence of MleR or L-malate [16]. Recently Sheng and Marquis showed that *S. mutan*s possesses MLF activity with a pH optimum of pH 4 in planktonic cells [17]. Significant intracellular ATP maintenance and enhanced protection against lethal pH values were observed in the presence of L-malate [17]. Since this study showed that MLF has a great impact on the aciduric capacities of *S. mutans*, we were interested if this mechanism is part of the general ATR of the cell or if it is specifically induced by MleR and the presence of L-malate. Deletion of *mleR *and luciferase reporter strains for *mleR *and *mleS *and RT-PCR revealed insights into the expression and regulation of the *mle *gene cluster and especially the effect of pH. Electrophoretic mobility shift assays (EMSA) indicated several binding sites for the MleR protein which were influenced by the presence of L-malate. Moreover we investigated the role of MleR for the ability of *S. mutans *to withstand acid stress.

## Results

### Analysis of the *mle *locus by RT-PCR and EMSA

**Figure 1:**
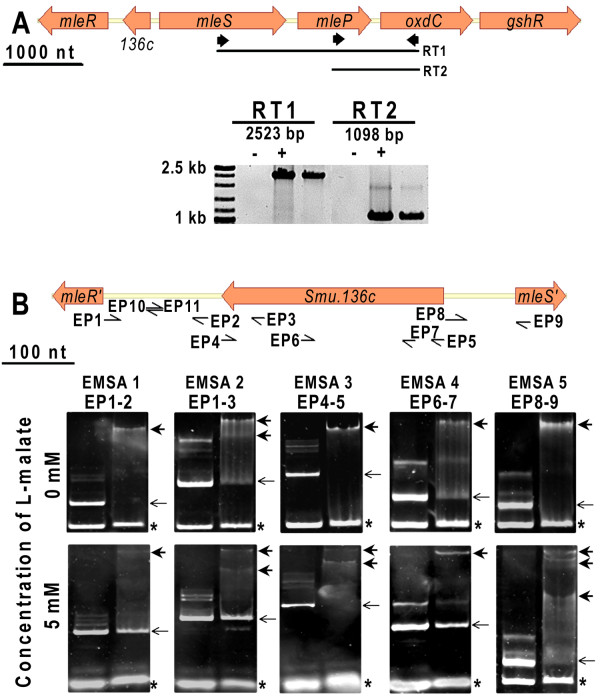
**Genetic organization of the *mle *locus**. A: RT-PCR analysis of mRNA transcripts. The solid arrows indicate the primers used for RT-PCR. The minus RT control is assigned with "-"; the positive control, using genomic DNA, is assigned with "+". B: Gelshift analysis of the region between *mleS *and *mleR*. Arrows indicate primers that were used to amplify PCR products, that were subsequently used for EMSA. Primers are designated at their 5' end. The box shows a representative selection of gel shift assays with the respective fragment in the presence or absence of L-malate. Thin arrows indicate DNA fragments in the absence of protein. Bold arrows indicate DNA in complex with MleR. Competitor DNA is marked with an asterix. For all EMSAs, 1× binding buffer was loaded on the left and MleR protein on the right lane. In all EMSAs without malate, an internal fragment of *mleS *was used as competitor DNA. In EMSAs with malate the fragment within the IGS of *mleR *and Smu.136c, generated by hybridising primers EP10/11 was used (except for EMSA 5, where the internal fragment of *mleS *was added).

In the genome of *S. mutans *UA159 [14], the lysR type transcriptional regulator MleR is orientated opposite to a gene cluster encoding the malolactic enzyme (*mleS*), a malate permease (*mleP*), and a oxalate decarboxylase (*oxdC*), respectively. Additionally a putative prophage repressor is inserted between *mleR *and *mleS *(Figure 1). This insertion is unique for the oral *streptococci *(*S. mutans *UA159, *S. gordonii *str. Challis CH1 and *S. sanguinis *SK36) among all sequenced *Lactobacillales*. Adjacent to the genes involved in malolactic fermentation is the gene *oxdC *encoding the oxalate decarboxylase which catalyses the conversion of oxalate to formate and CO_2_. This gene is unique for *S. mutans *UA159 among all sequenced *Lactobacillales*. RT-PCR disclosed that it is co-transcribed with *mleS *and *mleP *since it was possible to amplify overlapping fragments of all three genes (Figure 1A). The putative gluthatione reductase (Smu.140) located downstream of *oxdC*, which is involved in the removal of reactive oxygen species, could not be assigned to the same operon by the use of RT-PCR.

Applying the Electrophoretic Mobility Shift Assay (EMSA) (Figure 1B) using a DNA fragment covering almost the complete intergenic sequence (IGS) between *mleR *and Smu.136c (EMSA 1) resulted in one retarded complex, indicating one binding site for MleR in this intergenic region. Elongation of the DNA fragment (EMSA 2) to include the 3' end of Smu.136c, produced two retarded bands, suggesting an additional binding site at the 3' end of Smu.136c. The presence of 5 mM L-malate in both EMSA reactions gave the same banding pattern. However, the extent of the shift was slightly reduced.

Using the complete coding sequence of Smu.136c (EMSA 3) resulted in one retarded complex, confirming the presence of one binding site for MleR in this gene. Addition of L-malate to the binding reaction changed the pattern in this case and produced two retarded fragments. Truncation of the 3' end of Smu.136c (EMSA 4) resulted only in one retarded fragment, independent of L-malate. The data show the presence of at least two binding sites for MleR within Smu.136c. One site is located within fragment EP 6-7 (EMSA 4) presumably binding the *apo *form of MleR and another one is located at the 3'end of Smu.136c and appears to need the co-inducer bound form of MleR. The intergenic sequence upstream of *mleS *(EMSA 5) produced one retarded complex in the absence and three complexes in the presence of 5 mM L-malate. Thus, within this IGS also several binding sites for different forms of MleR exist. Using internal DNA fragments of *mleS *or *mleR *(data for *mleR *not shown) or a sequence within the IGS of *mleR *and Smu.136c (primers 137qF/R) did not produce complexes with the MleR protein under the tested condition, thus confirming the specificity of the DNA-protein interaction. Incubation of all used DNA fragments with BSA instead of MleR resulted in no retardation (data not shown).

### Involvement of *mleR *in MLF activity

It was previously shown that *S. mutans *UA159 was able to carry out malolactic fermentation [17]. To determine if the putative regulator MleR is involved in the regulation of the MLF gene cluster a knockout mutant of *mleR *was constructed, by replacing an internal part (amino acids 27-275) of the gene with an erythromycin resistance cassette, amplified from another strain [18]. *S. mutans *wildtype cells showed highest MLF enzyme activity in the presence of 25 mM L-malate at the beginning of the stationary phase [17]. Under these conditions, we observed a significant reduction of MLF activity of the Δ*mleR *mutant compared to the parental strain, indicating a positive regulation of the *mle *genes by MleR (Table 1). After one hour the wild type strain converted or internalised over 40% of the added L-malate. For the mutant lacking the MleR regulator only a 1% reduction of the added malate within one hour was observed. Furthermore, internalisation and decarboxylation of the stronger malic acid to lactic acid leads to a considerable increase of the external pH (Table 1). However, after 12 hours of incubation a reduction of 78% and 38% of the added L-malate was observed in the wildtype and the Δ*mleR *mutant, respectively, indicating a basal level of MLF enzyme activity in the absence of MleR.

**Table 1:** Malolactic fermentation activity for the wildtype and the Δ*mleR *mutant.

	L-malate concentration [mg/ml]		pH-value	
	
Time	WT	Δ*mleR*	WT	Δ*mleR*
0 min	5.53	5.63	6.4	6.34
20 min	4.87	5.61	6.7	6.32
40 min	2.77	5.59	6.9	6.43
60 min	2.34	5.42	7.2	6.52
12 hours	1.26	3.51	8.2	7.32

The capability to carry out malolactic fermentation was determined by measuring the L-malate concentration and the pH of the supernatant of cultures grown to late exponential phase (OD ~1.3). The values represent the average of two independent experiments. The standard deviation was less than 5%.

### Transcription of *mle *genes during growth

**Figure 2:**
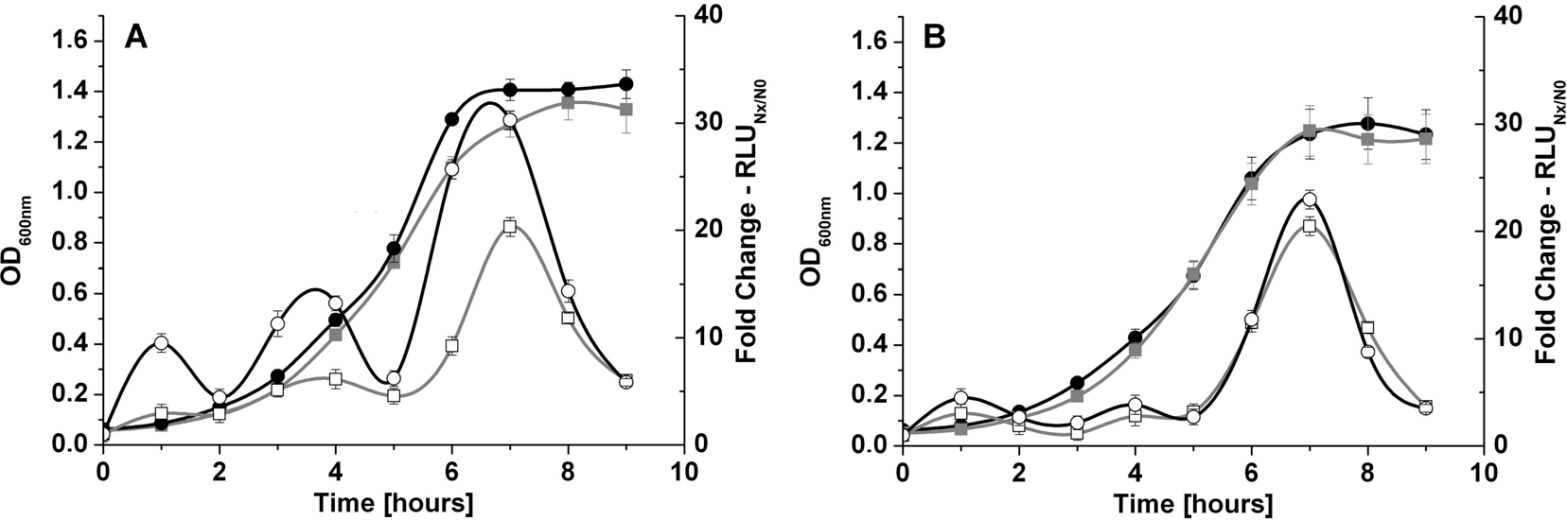
**Promoter activity of *mleR *and *mleS *in the absence of malate**. Promoter activity of *mleR *and *mleS *during batch cultivation in BMS medium without malate under anaerobic conditions. A: Optical density and luciferase activity of both promoters in the wildtype background. B: Optical density and luciferase activity of both promoters in the Δ*mleR *background. Grey square, Optical density of strains carrying *mleR*_p_-luc; Black circle, Optical density of strains carrying *mleS*_p_-luc; Open square, RLU of strains carrying *mleR*_p_-luc; Open circle, RLU of strains carrying *mleS*_p_-luc

To obtain better insights into the transcriptional regulation of the MLF gene cluster and *mleR *itself, firefly luciferase reporter plasmids were constructed. The upstream sequences of *mleR *and *mleS *containing the putative promoter sequences were cloned in front of a promoterless luciferase gene and then integrated into the genome of the wildtype and the Δ*mleR *mutant by single homologous recombination, respectively. Luciferase activity was monitored during growth in the absence of L-malic acid (Figure 2). The highest activity for both promoters was observed at the transition to the stationary phase, with an external pH between 5.8 and 6.1. This was true for the parental strain and the Δ*mleR *mutant, indicating that both transcriptional units might be controlled by acid inducible promoters. To rule out that this up-regulation was not due to post-exponential phase phenomena, we investigated the influence of the pH during the exponential growth phase in more detail (see below). However, in the wildtype the *mleS *promoter construct showed higher activity than in the Δ*mleR *knockout strain, indicating that MleR induces transcription even in the absence of the potential co-inducer L-malate. Accordingly, quantitative real time RT-PCR of RNA isolated from cells in the late exponential phase in the absence of L-malate showed a 3-fold induction of the genes *mleS *and *mleP *when comparing the wildtype to the Δ*mleR *mutant strain. An induction of *mleR *itself under these conditions was not observed (data not shown).

### Regulation of the *mle *genes by pH and L-malate

**Figure 3:**
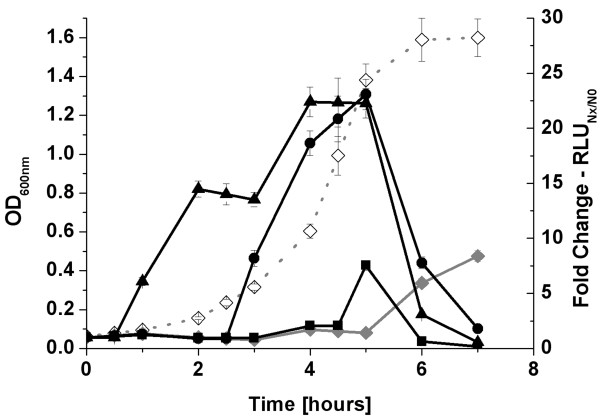
**Promoter activity of *mleR *in the presence of malate**. Influence of L-malate (25 mM, not neutralized) on the promoter activity of wildtype *S. mutans *carrying *mleR*_p_-luc in BMS medium under anaerobic conditions. Open diamond, growth without malate; Grey diamond, RLU, no addition of L-malate; Triangle, RLU, addition of L-malate after 30 min; Circle, RLU, addition after 2.5 hours; Square, RLU, addition after 4.5 hours.

**Figure 4:**
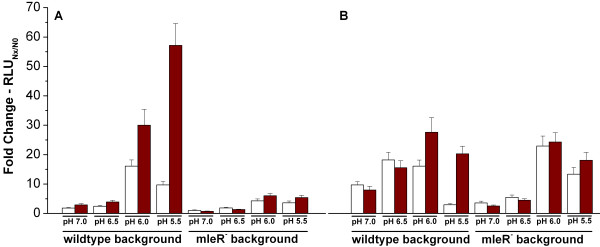
**Influence of pH and L-malate on promoter activity of *mleR *and *mleS***. Cells of wildtype and Δ*mleR *were cultivated in BMS under anaerobic conditions. Neutralized L-malate was added to the respective samples and the pH was adjusted to the desired values. A: Fold change of RLU after two hours of strains carrying *mleS*_p_-luc. Left, wildtype. Right, Δ*mleR *mutant. B: Fold change of RLU after two hours of strains carrying *mleR*_p_-luc. Left, wildtype. Right, Δ*mleR *mutant. White bars, no addition of L-malate; Red bars, addition of 25 mM L-malate.

During batch cultivation without addition of external L-malate, the highest luciferase activity for the *mle *promoters was observed during the transition to the stationary phase (see above). Addition of L-malate as free acid to the culture (end concentration of 25 mM), thereby lowering the pH to 5.6-6.2 (depending on the growth stage in BM medium), resulted in an immediate induction of activity (Figure 3). To determine if this effect was caused by the low pH or by L-malate, we further studied the influence of both parameters separately. After inoculation, cells were allowed to adapt for two hours to the medium. After addition of neutralized L-malate (25 mM final concentration) the pH of the cultures was adjusted with HCl to the desired values and samples for luciferase measurements were withdrawn in intervals of 30 min for two hours. Figure 4 summarizes the fold change values of promoter activity after two hours of measurement. Lowering the pH, without addition of malate, resulted in an increased activity of both promoters in the wildtype as well as in the *ΔmleR *background. These data clearly demonstrate that both promoters are acid inducible and that this behaviour was not caused by post-exponential phenomena. Furthermore, it shows that the influence of MleR is weak at neutral pH conditions. By contrast, the presence of L-malate at low pH significantly enhanced the activity of both promoters, but only in the presence of a functional copy of *mleR*. This allows four conclusions: (a) L-malate is the coinducer of MleR; (b) enhanced transcription in the presence of L-malate requires an acidic pH; (c) MleR positively regulates its target genes and furthermore (d) its own transcription. A positive auto-regulation would be a special feature, since most LTTR repress their own transcription. However, exceptions exist e.g. LrhA [19]. However, no significant induction of *mleR *after two hours exposure to 25 mM free malic acid was observed using quantitative real time PCR (See below).

### Quantitative real time PCR

**Figure 5:**
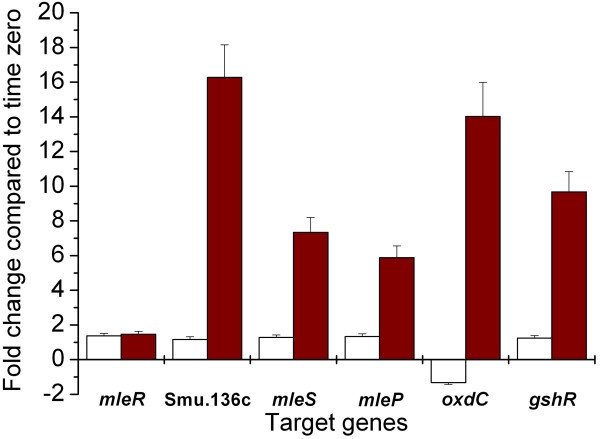
**Induction of the *mle *locus by low pH and malate**. The transcription level was determined by quantitative real time RT-PCR of the genes Smu.135-140. Results are presented as fold change after a two hours treatment with 0 or 25 mM L-malate and compared to time zero. White bars, 0 mM free malic acid; Red bars, 25 mM free malic acid.

The transcript levels of the genes Smu.135-140 were determined using quantitative real time RT-PCR. To this end, an early log phase culture of the wildtype was divided. To one part free malic acid (25 mM final concentration) was added, the other part remained untreated. RNA was sampled prior to splitting the culture and after two hours. All tested genes, except *mleR *itself, showed enhanced transcription in the presence of malic acid compared to time zero (Figure 5).

### Influence of L-malate and MleR on growth

**Figure 6:**
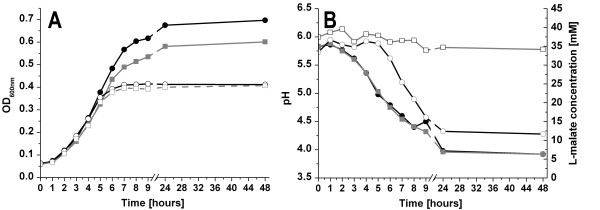
**Influence of L-malate and *mleR *on the growth of *S. mutans***. Cell were inoculated in acidified BMG (pH 6.0) medium under anaerobic conditions. A: Growth (OD_600_) of wildtype (black) and Δ*mleR *mutant (grey) in the absence (open symbols) or presence (filled symbols) of L-malate. B: pH and malate concentration of the supernatant of wildtype and Δ*mleR *mutant cultures grown in the presence of malate. Closed circle, pH of wildtype; Closed square, pH of the Δ*mleR *mutant; Open circle, malate concentration of wildtype; Open square, malate concentration of the Δ*mleR *mutant.

Since L-malate does not serve as a catabolite facilitating growth of *S. mutans *we were interested to see how energy gain and pH maintenance due to MLF affect its ability to grow in an acidic environment. To study this, we used BM medium supplemented with 1% (w/v) glucose (pH adjusted to 6.0) with or without supplementation of L-malate. In the absence of L-malate, there was no difference in growth of the wildtype and the Δ*mleR *mutant strain. Both strains entered the stationary phase after 6-7 hours at an external pH of about 4.2 and reached a final OD_600 _of about 0.41 (Figure 6A). Inoculation of neutral BMG with this culture (pH 7.4) resulted in an optical density of ~ 1.0 for both strains, ensuring that the pH and not nutrient limitation were the determinant for entering the stationary phase at acidic conditions. Addition of L-malate to the acidified culture medium facilitated pH maintenance and further growth of both cultures (Figure 6A). The presence of L-malate resulted in a substantially higher optical density of the wild type compared to the *mleR *knockout strain. Both strains were capable of carrying out MLF, as monitored by the L-malate concentration in the supernatant (Figure 6B), but the mutant to a much smaller degree than the wildtype. Further on significant internalisation/decarboxylation of L-malate started when the external pH dropped below 5, confirming the luciferase reporter data which had shown that the malolactic fermentation system is only activated at low pH.

### Influence of L-malate and *mleR *on the ability of *S. mutans *to tolerate acid stress

**Figure 7:**
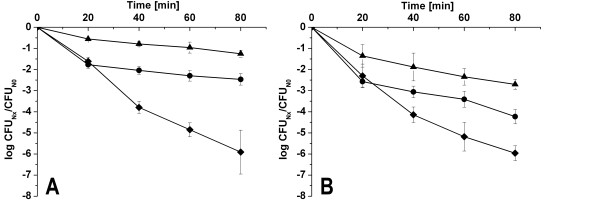
**Acid tolerance assay**. Role of malate for the survival of *S. mutans *wildtype (A) and Δ*mleR *mutant (B) after acid stress. Diamond, control, cells were incubated in neutral THBY without malate and subjected to pH 3.1 without malate; Circle, cells were incubated in neutral THBY without malate and subjected to pH 3.1 with malate; Triangle, cells were incubated in acidified THBY with malate and subjected to pH 3.1 with malate.

Since MLF has been shown to facilitate pH maintenance [17], we studied the contribution of MLF to acid tolerance in *S. mutans *(Figure 7). Control cells of wildtype and *ΔmleR *were grown in neutral THBY before being transferred to pH 3.1 without L-malate. Both strains showed no difference in the survival under these conditions (Figure 7). To determine the influence of malate and the *mleR *regulator on the response of *S. mutans *to a rapid pH shift, both the wildtype and the *mleR *mutant were grown in neutral THBY and then subjected to pH 3.1 in the presence of 25 mM malate. In both strains the number of surviving cells after 20 minutes was similar to the control (Figure 7). However, after 40 minutes the number of viable cells increased significantly compared to the control in the wildtype. Thus, the genes for MLF were induced within this time period and the conversion of malate contributed to the aciduricity. Without a functional copy of *mleR*, the number of viable cells also increased after 40 minutes but to a much lesser extend compared to the wildtype. This again shows that a shift to an acidic pH is satisfactory to induce the MLF genes in the absence of *mleR*. When the *mle *genes were induced by low pH and L-malate in a preincubation step before transferring the cells to pH 3.1, an immediately increased viability was already seen 20 minutes after acid shock. Again, the wildtype exhibited a significantly enhanced survival compared to the *mleR *knockout mutant. The data show that the MLF genes are induced during the acid adaptation response but a functional copy of *mleR *in conjunction with its co-inducer L-malate is needed to achieve maximal expression.

Quantitative real time PCR showed an up-regulation of the adjacent gluthatione reductase upon the addition of 25 mM free malic acid (Figure 5). Therefore, we tested the capability of *S. mutans *to survive exposure to 0.2 (v/v) hydrogen peroxide after incubation of cells in acidified THBY and malate to induce this gene. However, no difference between wildtype and Δ*mleR *mutant was observed (data not shown).

## Discussion

The aciduric capacity of *S. mutans *is one of the key elements of its virulence. Contributing mechanisms are increased activity of the F1F0-ATPase, changes in the membrane protein and fatty acid composition, the induction of stress proteins and the production of alkaline metabolites [10,20-22]. Extrusion of protons via the F1F0-ATPase consumes energy in the form of ATP. Hence, the yield of glycolytic activity and ATP production is diminished at low pH, *S. mutans *has to induce other pathways to supply enough energy. The conversion of L-malate to L-lactate and carbon dioxide during malolactic fermentation facilitates the maintenance of the ATP pool of the cell and supports the production of more alkaline metabolites. Therefore MLF directly contributes to the competitive fitness of *S. mutans *in the complex, multispecies environment of the dental plaque. Recently, Sheng and Marquis showed that cells of *S. mutans *UA159 possess MLF activity but no information about its regulation was available [17]. According to the information of MLF from *L. lactis *it was likely that the LTTR *mleR *adjacent to the MLF genes might be involved in their regulation.

### Low pH is required for induction of MLF

A knockout of *mleR *significantly decreased MLF activity of *S. mutans *cells and thus confirmed its participation in the regulation of MLF. Applying promoter luciferase reporter constructs we showed that the regulation of the *mle *genes is much more complex than just being induced in the presence of MleR.

The luciferase fusion data and the acid killing profiles showed that the *mle *genes are activated within 30 minutes by acidic pH values, independently of MleR and malate. Therefore, the transcription of the *mle *genes is driven from acid inducible promoters and MLF is part of the early acid tolerance response. The EMSA experiments showed a clear interaction of MleR with malate, even under alkaline conditions. However, under neutral pH conditions no effect of malate on the transcription (using the luciferase reporters) was noticeable, suggesting that uptake of malate occurs only under low pH conditions. Indeed, Poolman *et al*. [12] showed that in the presence of a pH gradient, membrane vesicles of *L. lactis *are able to take up L-malate with one proton or the monoanionic form of L-malate (MH^-^). They conclude that a pH gradient stimulates indirectly a malate/lactate antiport, by affecting the L-lactate gradient or promotes directly electrogenic malate uptake, respectively. They showed that with decreasing pH, the pH gradient adjusted to the membrane potential or even exceeded it, which resulted in an increased uptake of added malate. Assuming a similar mechanism in *S. mutans *explains why malate under neutral pH conditions did not cause an induction of the *mle *genes. Since the uptake of malate is reduced in a neutral pH environment, the intracellular amount of malate is not sufficient to stimulate MleR and subsequent avoided a positive regulation.

MleR fully induces the MLF only at low pH, with malate acting as a coinducer. A similar mechanism was recently disclosed by Liu *et al*. for the agmatine deiminase system [23]. They showed that its induction by AguR requires both low pH and agmatine. Using a linker scanning mutagenesis approach they were able to isolate mutant forms of AguR that lost their ability to activate transcription in response to pH, agmatine or both signals, respectively. They suggested that acidic conditions favoured binding of the ligand due to conformational changes of the regulator protein. A similar mechanism may indeed also be true for MleR and L-malate.

In *S. mutans*, MLF is switched on at low pH in the complete absence of malate. This behavior might be adaptive since low pH and the availability of malate are often correlated in natural sources, e.g. fruits. Thus, it may be advantageous for *S. mutans *to induce the whole battery of acid tolerance responses if threatened by low pH in order to be prepared, since chances of encountering malate are usually high.

### The mle locus

By RT-PCR we showed that the oxalate decarboxylase gene (*oxdC*) is co-transcribed with the *mleSP *genes. Since the reactions catalysed by MleS and OxdC are analogous it can be expected that decarboxylation of oxalate to formate also contributes to the aciduricity of *S. mutans*. However, no evidence for oxalate decarboxylation activity was found in *S. mutans *under the tested conditions, but extensive investigations were not carried out. Examination of the transcript levels of the wildtype in the presence of free malic acid using quantitative real time PCR showed co-transcription of *oxdC *with the *mle *genes and confirmed the results obtained with the luciferase reporter strains. The transcript level of *mleR *itself constituted an exception because it was not elevated. However, the result has to be interpreted cautiously since the reporter strains used here do not take into account the mRNA stability of *mleR*, which might represent another regulatory mechanism. Furthermore qPCR showed an induction of the adjacent gluthatione reductase, confirming that the responses to acidic and oxidative stress are overlapping in *S. mutans *[24].

### MleR binding sites

The electrophoretic mobility shift assays shown here revealed the presence of multiple binding sites for MleR in the DNA region within the translational start site of *mleR *and *mleS*. LysR type transcriptional regulators (LTTR) are generally regarded to be active as tetramers, therefore they are known to interact with several binding sites at their promoter region(s). The (auto)-regulatory binding site is favoured by the apo-form, whereas the (target)-activation site is occupied once the co-inducer is bound to the protein. However, the presence of the co-inducer affects the affinity to each binding site, influences DNA bending and subsequently protein-protein interactions [25,26].

The addition of L-malate changed the retardation pattern for some of the applied DNA fragments. Since the transcription of *mleR *and *mleS *was shown to be induced equally by a pH shift and L-malate using the luciferase reporter strains, a similar retardation behaviour in the EMSA for both upstream DNA fragments would have been expected.

Surprisingly, only the IGS upstream of *mleS *showed a different pattern in the presence of malate, whereas the IGS upstream of *mleR *even showed a weaker retardation. Due to the basic pI of the MleR protein, we were not able to carry out EMSA under physiological pH conditions which might negatively influence the binding affinity of the protein. The presence of at least two binding sites for MleR within the coding region of Smu.136c suggests a complex regulatory mechanism, which has to be elucidated further by means of DNase footprinting and mutagenesis.

## Conclusion

In summary, we showed that the *mle *genes including *oxdC *are under the control of acid inducible promoters and that they are induced within the first 30 minutes upon acid shock. Therefore they are part of the early acid tolerance response in *S. mutans*, which is induced within 30 minutes after acidification [8]. Further enhancement of their transcription can be obtained by MleR and L-malate in an acidic environment. The use of gel retardation assays showed the presence of multiple binding sites for MleR, even in the coding sequence of another gene, suggesting a complex regulatory mechanism. We clearly showed that the presence of L-malate contributed strongly to the survival of *S. mutans *under low pH conditions. MLF is one of the strategies aciduric bacteria have evolved to cope with low pH and to compete with other bacteria in dental plaque. *S. mutans *is able to carry out MLF under more acidic conditions than other *Streptococci *[17], thus emphasizing the dominant role of *S. mutans *in the oral cavity.

## Methods

### Bacterial strains, plasmids, and culture conditions

Bacterial strains and plasmids and their relevant characteristics are listed in table 2. *Escherichia coli *was routinely cultured in Luria Bertani (LB, Carl-Roth, Karlsruhe, Germany) medium at 37°C. *E. coli *strains carrying plasmids were selected with 100 μg ml^-1 ^ampicillin, or 50 μg ml^-1 ^spectinomycin. All *Streptococcus mutans *strains were cultivated in Todd Hewitt Broth medium supplemented with 0.1% (w/v) yeast extract (THBY, Becton Dickinson, Heidelberg, Germany) or in BM [27] medium containing 0.5% sucrose (BMS) or 1% (w/v) glucose (BMG). *S. mutans *strains were grown at 37°C without agitation aerobically (5% CO_2 _enriched) in THBY or in BM medium under anaerobic conditions (80% N_2_, 10% H_2_, 10% CO_2_). Pre-cultures were grown in THBY medium. Selection of mutant strains was carried out with 10 μg ml^-1 ^erythromycin, or 500 μg ml^-1 ^spectinomycin.

**Table 2:** Bacterial strains and plasmids used in this study.

Strain/plasmid	Relevant Characteristics^a^	Reference/source
Strains		
*E. coli*		
DH5α	General cloning strain	
Tuner(DE3)	Expression strain	Novagen
		
*S. mutans*		ATCC 700610
UA159	Wild-type, Erm^s^, Sp^s^	This study
ALSM3	UA159Δ*mleR*, Erm^r^	This study
ALSM20	UA159::ϕ(*mleR*_P_-luc), Sp^r^	This study
ALSM13	UA159Δ*mleR*::ϕ(*mleR*_P_-luc), Erm^r^, Sp^r^	This study
ALSM33	UA159::ϕ(*mleS*_P_-luc), Sp^r^	This study
ALSM34	UA159Δ*mleR*::ϕ(*mleS*_P_-luc), Erm^r^, Sp^r^	This study
		This study
		This study
Plasmids		
pFW5	Suicide vector, Sp^r^	A. Podbielski [29]
pHL222	Ap^r^, luc	H. Lössner
pALEC15	Derivate pFW5, luc, Sp^r^	This study
pALEC16	pALEC15 + ϕ(*mleR*_P_-luc), Sp^r^	This study
pALEC47	pALEC15 + ϕ(*mleS*_P_-luc), Sp^r^	This study

^a ^Ap^r^, ampicillin resistance; Sp^r^, spectinomycin resistance; Erm^r^, erythromycin resistance; luc, luciferase.

### Construction of *mleR *knockout mutant

The null mutant of *mleR *(Smu.135) was constructed by allelic replacement using the PCR ligation mutagenesis strategy described by Lau *et al*.[28]. To generate the construct, two fragments upstream and downstream of the *mleR *gene were amplified with *Pfu *polymerase (Promega) with primers 135UpF/135UpR and 135DoF/135DoR (Table 3). Restriction sites were incorporated into the primers and the amplicons subsequently digested with the appropriate enzyme. The erythromycin antibiotic resistance cassette was amplified with primers ermF/ermR and treated as described above. All fragments were ligated and transformed into *S. mutans *UA159 to generate strain ALSM3 as previously described [18]. Erythromycin resistant colonies were confirmed by PCR and sequencing.

**Table 3:** Primers used in this study.

Primer^a^	Sequence (5'→3')	Purpose
135UpF	CCAAATAACCCGCATATTGAGG	Knockout *mleR*
135UpR	**GGCGCGCC**TTGAAATTTTTCAGCAACCTTA	Knockout *mleR*
135DoF	**GGCCGGCC**TCCTCAACCTTAACACCTGATA	Knockout *mleR*
135DoR	GTTGCTAAAGATTTGTTCTCAG	Knockout *mleR*
ErmF	**GGCGCGCC**CCGGGCCCAAAATTTGTTTGAT	ErmEA
ErmR	**GGCCGGCC**AGTCGGCAGCGACTCATAGAAT	ErmEA
lucF	ATATA**CCATGG**AAGACGCCAAAAAC	Luciferase
lucR	AAAAAA**ACTAGT**TTATGCTAGTTATTGCTCAGCGG	Luciferase
P135F/EP9	AAAAAA**CCATGG**CTTTATTCAAAAAAGGATCGTTT	Promoter *mleR*/EMSA
P135R	TTTTTT**CCATGG**TTAACCTTTCTATTATTTTTACTAGTT	Promoter *mleR*
P137F/EP6	AAATTT**CCATGG**CAAGACTGTTAAAGTCAAAAA	Promoter *mleS*/EMSA
P137R/	AAAAAA**CCATGG**TTTCTGCACCTCCTTATATT	Promoter *mleS*
135qF	TGAAGCGTCACCTTGAGAGA	Smu.135 QPCR
135qR	TAATGGGTGGGCATCCTAAG	Smu.135 QPCR
136qF	AAGGTATCATCGGCAAGCAC	Smu.136 QPCR
136qR	TCACTTTTTCAAGCGTCTGC	Smu.136 QPCR
137qF	GGTATCTTTGCGGCTATGGA	Smu.137 QPCR
137qR	TTTCACGCAAGACACGAGAG	Smu.137 QPCR
138qF	CGACGGATAGCAAGTCTGGT	Smu.138 QPCR
138qR	GTCAACGTGCTAGTCGCAAA	Smu.138 QPCR
139qF	TACAGCGATTGACGAGAACG	Smu.139 QPCR
139qR	AGAAATTGGCTTCGCTGAAA	Smu.139 QPCR
140qF	TTCCTATGCGGATTTTCAGG	Smu.140 QPCR
140qR	CCTGACCGATTTGGGAATA	Smu.140 QPCR
1114qF	TACTACCCGGCCCCGATT	Smu.1114 QPCR
1114qR	CGAGCACGCAAAACAATAGA	Smu.1114 QPCR
EP1	TTAACCTTTCTATTATTTTTACTAGTT	EMSA
EP2	TCCAAGTGGTTTAAAAGTAACAAGA	EMSA
EP3	GCAACTTCCCAAGAGAAAACA	EMSA
EP4	TTAATCAAGATTATCAATAATCTC	EMSA
EP5	ATGAAGAAAAAAAGCTATCT	EMSA
EP7	TGCTTGCCGATGATAGGTT	EMSA
EP8	TAAAGAATACAAGTTTAAAAGCAAATAGTTAACT	EMSA
EP10	ATAAGTATTTTTTATCCGTTATCTAAGGTTTGAC	EMSA
EP11	GTCAAACCTTAGATAACGGATAAAAAATACTTAT	EMSA

^a ^Restriction sites in bold

### Construction of luciferase reporter strains

For the construction of the luciferase reporter strains, the advanced firefly luciferase was amplified using *Pfu *polymerase from plasmid pHL222 using primers lucF/lucR. The amplicon was cloned into the suicide vector pFW5 [29] via the NcoI and SpeI sites to generate plasmid pALEC15. The upstream regions containing the putative promoters of *mleR *and *mleS *were amplified using the primers P135F/P135R and P137F/P137R. The PCR products were digested with NcoI and ligated into pALEC15 to generate plasmids pALEC16 and pALEC47, respectively. The plasmids were transformed into the wildtype and strain ALSM3 to generate strains ALSM20, ALSM13, ALSM33, and ALSM34.

### Luciferase assay

Luciferase assays were performed by withdrawing 1 ml culture. The OD_600 _was measured and samples were held on ice until the start of the assay. 100 μl of each sample were mixed with 3× assay buffer (75 mM tricine, 15 mM MgSO_4_, 1.5 mM EDTA, 1.5 mM DTT, 900 μM ATP, 3 mg/ml (w/v) BSA, and 3% (w/v) D-Glucose, pH = 7.8) and incubated 10 min prior to injection of 100 μl D-luciferin (120 μM final concentration) solved in 20 mM tricine (pH 7.8). D-Luciferin (Carl-Roth, Karlsruhe, Germany) was resuspended in 20 mM tricine (pH = 7.8, 1 mg/ml), aliquoted and stored at -70°C until use. Luminescence was recorded for 35 s (POLARstar OPTIMA luminometer, BMG LABTECH) and normalized against the OD_600 _to calculate the relative light units (RLU). For calculation of the fold change, the RLU were normalized against the RLU of time zero. All measurements were done in triplicate.

### RNA extraction and quantitative real-time RT PCR

*S. mutans *wildtype was incubated anaerobically in BM medium containing 0.5% (w/v) sucrose until early-log phase. A sample was withdrawn for time zero, transferred into the double volume of RNA-protect (Qiagen, Hilden, Germany) and centrifuged according to the manufacturer's instructions. The cultures were split in two halves and free malic acid was added to one of them (final concentration 25 mM). After two hours samples for RNA extraction were withdrawn and treated as described above. For lysis, cells were incubated with lysozyme (2.5 mg/ml culture pellet) and mutanolysin (50 U/ml culture pellet) at room temperature for 45 min. The mixture was transferred into RLT buffer containing sterile, acid washed glass beads (diameter 106 μm) and vortexed for 3 min. Subsequent RNA extraction was carried out using the RNeasy mini kit (Qiagen). Genomic DNA was removed using the DNAse I (Qiagen) in-solution digestion protocol. The quality of the total RNA was controlled on a denaturating formaldehyde agarose gel. Synthesis of cDNA was carried out using random hexamers and SuperScript II reverse transcriptase (Invitrogen, Karlsruhe, Germany), followed by purification using the PCR Purification kit (Qiagen). All reactions included a control without SuperScript II to assess genomic DNA contamination. Real-time PCR was performed using the LightCycler 480 system (Roche, Mannheim, Germany) and the reaction mixtures were prepared using the Quantitect SYBR Green PCR Kit (Qiagen). Changes in the level of gene expression were calculated automatically by the LightCylcer 480 software using the ΔΔ*C_T _*method. The gyrase A gene (Smu.1114) was used as the housekeeping reference gene. All steps were performed according to the manufacturer's protocols. All measurements were done in duplicate.

### Acid killing and hydrogen peroxide killing

The ability of *S. mutans *to withstand acidic pH was determined by the method of Belli and Marquis [9]. Briefly, overnight cultures of *S. mutans *strains were diluted 1:20 in fresh THBY medium (pH 7) and grown under aerobic conditions. Cultures were harvested (at an OD_600 _~ 0.3) by centrifugation at 11000 × *g *for 5 min. The supernatant was carefully discarded and the pellet was resuspended in 0.1 M glycine buffer pH 7.0 (time zero) or pH 3.1 without malate (control) or in the presence of 25 mM L-malate. Samples of cells incubated at pH 3.1 were withdrawn after 20, 40, 60, and 80 minutes, serially diluted in 0.1 M glycine buffer, pH 7.0, and plated on THBY plates in triplicate and incubated for 48 h aerobically.

For pre-induction of the acid tolerance response and to achieve maximal expression of MLF, cells were grown in THBY (pH 5.5) in the presence of 25 mM L-malate and treated as described above. To determine the capability to withstand hydrogen peroxide, cells were collected as described above and resuspended in 0.1 M glycine buffer, pH 7.0. Before the addition of H_2_O_2_, 0.2% (v/v) final concentration, an aliquot was withdrawn to determine the cell number by colony forming units at time zero. To inactivate hydrogen peroxide, catalase (5 mg/ml, Sigma) was added immediately after sampling. Samples were serially diluted in 0.1 M glycine buffer, pH 7.0, plated in triplicate and incubated as described above.

### Assay for malolactic fermentation activity

The capacity to carry out malolactic fermentation was determined by the method of Sheng and Marquis [17], slightly modified. Briefly, *S. mutans *cells were cultivated in THBY aerobically until the end of the log phase. An equal amount of wildtype and *ΔmleR *cells was harvested by centrifugation (5000 × *g*, 15 min, 4°C) washed with salt solution (50 mM KCl + 1 mM MgCl_2_) and incubated for 1 h in 20 mM potassium phosphate buffer, pH 7.0 at 37°C. The pH of the cultures was adjusted with HCl to pH 6.3. Prewarmed L-malate was added to the cell suspension (42 mM end concentration) to initiate malolactic fermentation. Aliquots were withdrawn after 0, 20, 40, and 60 minutes and 12 hours for measuring the pH and the L-malate concentration of the supernatant using the L-malic acid kit from Biosentec (Toulouse, France). For determination of L-malate in growing cultures, 1 ml was centrifuged at 11000 × *g *for 5 min and the supernatant was analysed using the L-malic acid kit.

### Expression and purification of the MleR protein

For expression the coding sequence of *mleR *was amplified using primers CDSMleRF/R and cloned into the pET28c expression vector (Novagen, Merck KgaA, Darmstadt, Germany) via the NdeI and NheI restriction sites. The resulting plasmid was sequenced for confirmation and further transformed into *E. coli *Tuner DE3 (Novagen) to obtain an N-terminal 6His fusion protein. For expression a 250 ml LB culture was grown to an OD_600 nm _of 0.6 and expression was induced by adding IPTG to a final concentration of 1 mM. The cells were grown for additional two hours, harvested (4,000 × *g*, 20 min, 4°C) and resuspended in 50 mM NaH_2_PO_4_, pH 7.0, 300 mM NaCl, 25 mM imidazole, and 5 mg/ml lysozyme and incubated on ice for 30 min. Subsequently, the cells were further lysed by sonification (4 × 1 min pulse, 1 min break, MS72 probe with 25% power; Bandelin Sonoplus HD2200, Berlin, Germany) and the soluble 6His-MleR extract was separated from insoluble cell material by centrifugation (25,000 × *g*, 30 min, 4°C). The 6His-MleR protein was then purified by IMAC affinity chromatography using Talon resin (Clontech, Saint-Germain-en-Laye, France). Bound protein was washed with 8 bed volumes 50 mM NaH_2_PO_4_, pH 7.0, 300 mM NaCl, 25 mM imidazole and eluted with 50 mM NaH_2_PO_4_, pH 7.0, 300 mM NaCl, 300 mM imidazole. The eluted 6His-MleR protein (purity >90% on an SDS PAGE) was always stored on ice and was verified by western blot (Anti His-tag antibody, Novagen) and N-terminal sequencing.

### Electrophoretic mobility shift assay (EMSA)

For binding studies, the purified MleR protein was dialysed four times against 1 liter 1× binding buffer (20 mM Tris, pH 7.5, 100 mM KCl, 2 mM EDTA, 10% glycerol) at 4°C for 12 hours using a 12-14 kDa cut-off dialysis bag (Medicell International Ltd., London, UK). Several fragments of the region between *mleR *and *mleS *were PCR amplified and directly used for gel retardation experiments (see Table 3 for primers). To verify the specificity of the DNA-MleR interaction each reaction mixture contained an equal amount of competitor DNA. Competitor DNA consisted either of an internal fragment of *mleS*, amplified by PCR (primers 137qF/R), or a DNA fragment within the upstream region of *mleR*, generated by hybridising complementary primers (EP10/11, Table 3). For this purpose, primers EP10/11 were mixed in equal molar ratios, denaturated by heating to 100°C and annealed by slowly cooling down to room temperature. DNA fragments, MleR protein (appr. 100 ng) and competitor DNA (in case of the complementary primers 75 ng/μl, final concentration) were mixed and incubated for 20 min at ambient temperature. To further exclude unspecific interactions, MleR was substituted with 100 ng BSA (Carl-Roth) and tested for each fragment. The reaction mixtures were subsequently loaded onto a 0.5× TBE, 4.5% polyacrylamide (37.5:1, acrylamide/bisacrylamide) gel. Since the MleR protein has a calculated pI of ~9, DNA in complex with MleR was hardly entering the gel using pH values below 9.2. Therefore the pH of the gel cast solution and electrophoresis buffer were adjusted to pH 9.45. L-malate was added to the binding reaction, the gel and the electrophoresis buffer (0.5× TBE) at 5 mM final concentration when needed. Electrophoresis was carried out at 10 V/cm at ambient temperature and the gel was stained using SYBR Gold (Invitrogen).

## Authors' contributions

AL planned and carried out the experiments and wrote the original manuscript. IW-D and HS participated in the design of the study and helped to draft the manuscript.

All authors read and approved the final manuscript.
